# ﻿Two new species of *Ocotea* (Lauraceae) with clustered leaves from the Uxpanapa region, Mexico

**DOI:** 10.3897/phytokeys.252.132953

**Published:** 2025-02-14

**Authors:** Leopoldo Hurtado-Reveles, Andrés Ernesto Ortiz-Rodriguez

**Affiliations:** 1 Instituto de Biología, Departamento de Botánica, Universidad Nacional Autónoma de México, 04510, Mexico City, Mexico Universidad Nacional Autónoma de México Mexico City Mexico

**Keywords:** Endemic, floristic refuge, Tehuantepec Isthmus, tropical rainforests, Veracruz

## Abstract

Two new species of *Ocotea* (Lauraceae) from Southern Mexico are described and illustrated. Based on their floral morphology, vegetative indument, and micromorphological attributes, both species are circumscribed within the *Ocoteahelicterifolia* group. Within this clade, the two species are morphologically similar to those with clustered leaves. Both species are endemic to the species-rich yet fragmented forests of the Uxpanapa region in Mexico, and therefore, their populations are threatened. Here, the morphological similarities of both species and their conservation status are discussed.

## ﻿Introduction

Lauraceae, with around 55 genera and ca. 3000 species, is one of the most ecologically important families of woody plants in tropical and subtropical forests (Rohwer 1993; Chanderbali et al. 2001). Members of the Lauraceae family pose taxonomic challenges for botanists due to the small size and complex internal structure of their flowers, the absence of local taxonomic treatments and the difficulty of following the available dichotomous keys (van der Werff 1991; van der Werff and Richter 1996; Rohwer et al. 2009; Liu et al. 2017; Rohde et al. 2017). Consequently, many herbarium specimens are undetermined, incorrectly determined, or without updated nomenclature. Furthermore, the current estimates of Lauraceae diversity in many tropical areas do not consider the latest botanical collections and exploration efforts, leading to an underestimation of its species richness (van der Werff 2020).

The genus *Ocotea* Aubl. is the most species-rich neotropical Lauraceae genera. It has a very broad concept and has been considered a catch-all genus for taxa that cannot be confidently placed in more clear-cut Lauraceae genera (Rohwer 1991; Lorea-Hernández 2002; van der Werff 2002). Moreover, phylogenetic analyses consistently show that *Ocotea* is a polyphyletic genus (Trofimov et al. 2019; Penagos-Zuluaga et al. 2021). Thus, within *Ocotea* several groups of species can be recognized (van der Werff 2002). In recent years, some of them have been elevated to the rank of genus based on phylogenetic and morphological evidence (Trofimov et al. 2019; Trofimov and Rohwer 2020).

One of the best-defined groups in *Ocotea* is the so-called *Ocoteahelicterifolia* group, which consists of around 40 Mexican and Central American tree species (Rohwer 1991, van der Werff 2002). It is characterized by the combination of mostly pubescent leaves, hermaphroditic flowers with spreading tepals, short-filamented and papillose stamens, anthers with locelli arranged in two superposed pairs, and fruit cupules with a single margin (van der Werff 2002). Additionally, a recent study revealed micromorphological characteristics supporting this delimitation; *Ocotea*helicterifolia group species share a rhombic-shaped stomatal complex and a narrowly lip-shaped aperture field of stomata (Trofimov and Rohwer 2018). Ecologically, a significant number of species are found in cloud forests at high altitudes, whilst only some species inhabit lowland tropical forests (Rohwer 1991; Lorea-Hernández and van der Werff 2002; van der Werff 2002; Lorea-Hernández 2005). Moreover, phylogenetic analyses suggest that the *Ocoteahelicterifolia* group is a sister clade to the genus *Damburneya* and is not related to other species of *Ocotea* (Trofimov et al. 2016; Penagos-Zuluaga et al. 2021).

During a field study in Southern Mexico, two new species of *Ocotea* were collected in the tropical rainforest of the Uxpanapa region. In the context of the genus, the two new species belong to the *O.helicterifolia* group and present a unique combination of morphological features, which allows them to be distinguished from any other species. These interesting species are herein described and illustrated, and their morphological relationships with other species of *Ocotea* are discussed.

## ﻿Materials and methods

We visited and reviewed the specimens of *Ocotea* deposited at the MEXU herbarium, Universidad Nacional Autónoma de México (UNAM). Also, we consulted the digitized type specimens available at JSTOR Global Plants (http://plants.jstor.org/). Thus, the new species were recognized using the unique combination of features criterion (Donoghue 1985) through comparisons with morphologically similar species and literature review (e.g. Rohwer 1991, van der Werff 2002). Finally, species descriptions were elaborated following the terminology presented in Hickey (1973).

We analyzed the stomata features of both species using a scanning electron microscope (SEM) at the Biodiversity Photography and Microscopy Laboratory (LANABIO) at UNAM. Leaf material for SEM was rehydrated with hot water for an hour and immersed in distilled water with a drop of biological soap to be subjected to an ultrasonic bath for two rounds of 90 seconds to remove debris. Rehydrated and clean samples were then rinsed in two soaks of distilled water. Then, samples were dehydrated gradually with alcohol (ethanol) solutions of 10, 30, 50, 70, 80, 90 and 100% for 30 minutes each. Finally, samples were critical-point dried using extra-dry CO_2_, mounted on metal sample holders, and covered with gold. Sample observation was conducted with a Hitachi SU1510® (Hitachi City, Japan) scanning electron microscope.

Based on all known localities for the new species, we assessed their conservation status by calculating their extent of occurrence (EOO) and their area of occupancy (AOO) using the GeoCAT tool (Bachman et al. 2011) and applying the IUCN Red List categories and criteria and the guideline (IUCN 2012; 2024).

## ﻿Results

### ﻿Two new species of *Ocotea* belonging to the *Ocoteahelicterifolia* group

**Figure 1.**
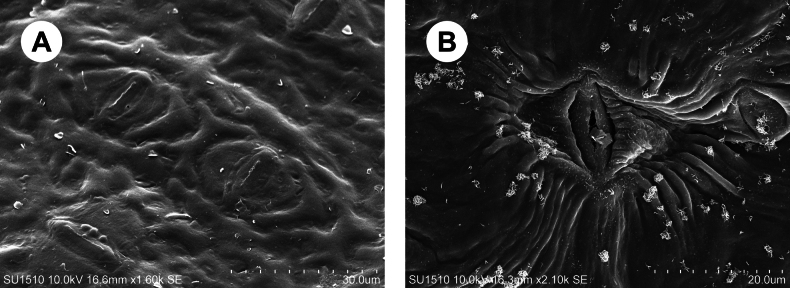
SEM photos of the stomatal complex **A***Ocoteabella***B***Ocoteacaelestis*.

The morphological characteristics of both species suggest that they belong to the *Ocoteahelicterifolia* group. Specifically, both species share pubescent leaves, hermaphroditic flowers with spreading tepals, short-filamented and papillose stamens and anthers with locelli arranged in two superposed pairs. This circumscription was further confirmed by their rhombic-shaped stomatal complexes (Fig. 1).

Within the *Ocoteahelicterifolia* group, both species are morphologically similar to *Ocoteabourgeauviana* (Mez) van der Werff, *Ocoteacongregata* van der Werff, *Ocoteatonii* (Lundell) van der Werff, and *Ocoteaverticillata* Rohwer, with which they share the clustered leaves. However, both species independently exhibit a unique combination of morphological characteristics, allowing us to conclude that they are new species for science (see Table 1). Thus, both species are described and illustrated below.

**Table 1.** Morphological differences among *Ocoteahelicterifolia* group species with clustered leaves.

Traits	*Ocoteabella*	*Ocoteacaelestis*	*Ocoteabourgeauviana*	*Ocoteacongregata*	*Ocoteatonii*	*Ocoteaverticillata*
Leaf size	13–30 × 3–8 cm	7–17 × 2–5 cm	10–22 × 3–7.5 cm	9–17 × 4–7 cm	15–25 × 5–6 cm	14–26 × 4.5–14.5 cm
Leaf apex shape	acuminate to caudate	acuminate to caudate	acuminate to caudate	obtuse to acute	acuminate	acuminate
Leaf base shape	acute to cuneate	acute to cuneate	cuneate	rounded or obtuse	rounded	obtuse to slightly cordate
Upper leaf surface texture	bullate	smooth	smooth	bullate	smooth	smooth
Petiole length	7–14 mm	5–8 mm	5–10 mm	10–25 mm	Up to 6 mm	3–8 mm
Flower diameter	5.5–6.5 mm	4–5 mm	5–6 mm	7–9 mm	5.5–6.5 mm	7–10 mm
Tepal outer surface	pubescent	glabrous	glabrous	pubescent	pubescent	pubescent towards the base
Tepal inner surface	pubescent towards the base	pubescent towards the base	pubescent	pubescent towards the base	pubescent	pubescent
Stamen color	reddish	white	White	white	white	white
Anthers	Without a sterile tip	With a sterile tip	Without a sterile tip	Without a sterile tip	With a sterile tip	With a sterile tip
Staminode shape	stipitiform	capitate	clavate	stipitiform	clavate	stipitiform

### ﻿Taxonomic treatment

#### ﻿ Ocotea bella

Taxon classificationPlantaeLauralesLauraceae

Reveles & Ortiz-Rodr. sp. nov.

CEEBCF0E-112F-54FE-BB16-963BF11BAC89

urn:lsid:ipni.org:names:77356682-1

Figs 2, 3

##### Type.

Mexico • Veracruz, Uxpanapa, 700 m southwest from Progreso Chapultepec village, 17°14'12.1"N, 94°18'23.4"W, 73 m, 14 Apr 2024, *Hurtado-Reveles L.* 1994 (holotype: MEXU; isotypes: NY, MO).

##### Diagnosis.

Similar to *Ocoteacongregata* in having clustered leaves with slightly bullate upper surface and condensed inflorescences, but differs from it in its narrowly elliptic to obovate leaves with acute to cuneate basis and shorter petioles, and by its smaller flowers with white tepals and reddish stamens (Table 1).

##### Description.

***Trees***, evergreen, 10–20 m tall, 15–20 cm DBH, bark smooth to slightly striate; twigs terete, densely covered with erect and adpressed, brown to light–brown trichomes, terminal buds 5–10 × 5–7 mm, densely covered with erect and adpressed golden sericeous trichomes. ***Leaves*** alternate-verticillate, clustered near the apex of branches (pseudo-verticillate), 3–8 × 13–30 cm, chartaceous, narrowly elliptic to obovate, apex acuminate to caudate, base acute to cuneate, often asymmetrical, upper surface slightly bullate, sparsely covered with erect light–brown trichomes, lower surface densely covered with erect light–brown trichomes, midrib slightly raised on the upper surface, prominently raised on the lower surface; secondary veins 9–12 on each side, slightly impressed above, prominently raised below; petiole swollen, 7–14 mm long, densely covered with erect and adpressed, brown to light–brown trichomes. ***Inflorescences*** condensed panicles, borne in the axils of distal leaves on recent growth, ca. 10 cm long, peduncles ca. 3 mm long, pedicels minute, the main axis, peduncles, pedicels and bracts densely covered with erect light–brown trichomes. ***Flowers*** bisexual, ca. 6 mm in diam.; tepals 6, subequal, white in anthesis, oblong, 2–2.5 × 1–1.5 mm, outer surface sparsely covered with adpressed, light–brown trichomes, inner surface glabrous or with trichomes only at the base; stamens 9, in three whorls, reddish in anthesis, ca. 1.5 mm long, filaments a third to half the length of the anther, densely covered with long hairs, as long as the filaments or as long as the stamens, anthers 4–locular, pollen sacs arranged in two pairs, one above the other, those in the outer two whorls introrse, those of the third whorl latrorse-extrorse, stamens of the third whorl with two globose glands at the base, staminodes minute, stipitiform, sometimes absent, pistil glabrous, ovary ovoid, 1 mm long, 0.6 mm in diam, style glabrous, ca. 0.1 mm long, stigma barely trifid. ***Fruits*** (immature), ellipsoid, 5–7 × 6–8 mm, cupule trumpet-shaped, 15–20 × 6–9 mm, some tepals persistent.

**Figure 2.**
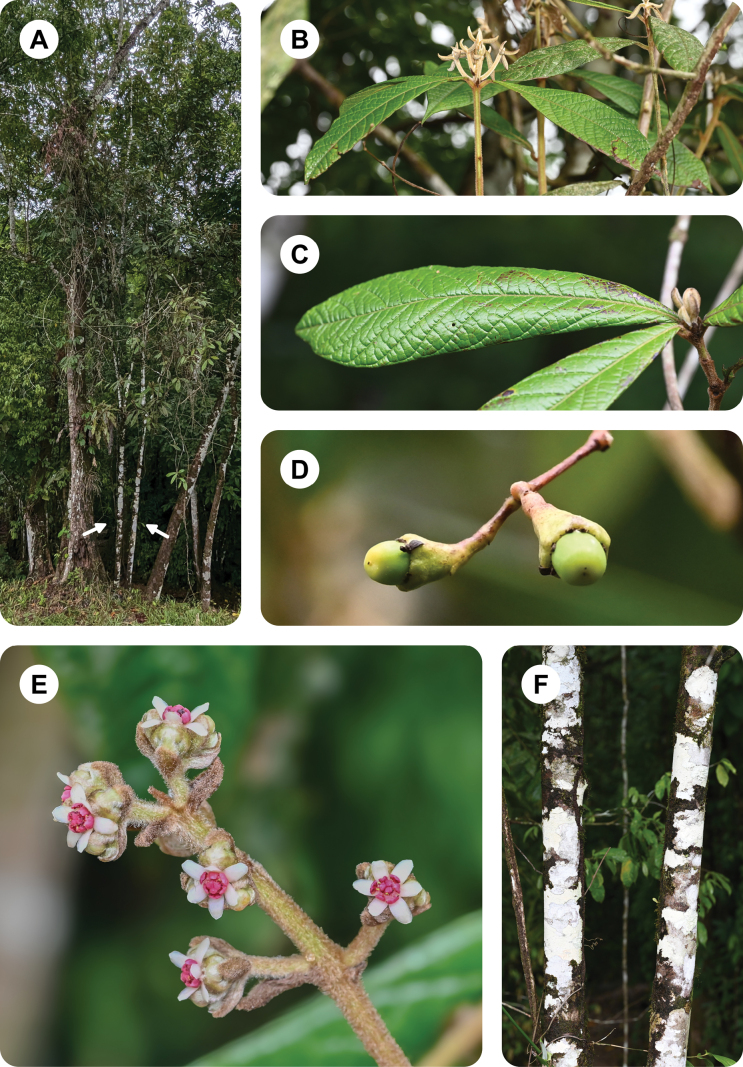
*Ocoteabella* Reveles & Ortiz-Rodr **A** general view of the tree (arrows indicate the specimen, which is a single individual with a bifurcated trunk at the base) **B** distal part of twig, with leaves and young growth **C** upper leaf surface **D** young fruits **E** inflorescence **F** trunks.

##### Phenology.

This species has been found with flowers in April and with fruits in June and July.

##### Etymology.

The specific epithet refers to the remarkable beauty (from Latin *bella* = beautiful or pleasant) of the flowers and leaves of this species.

**Figure 3.**
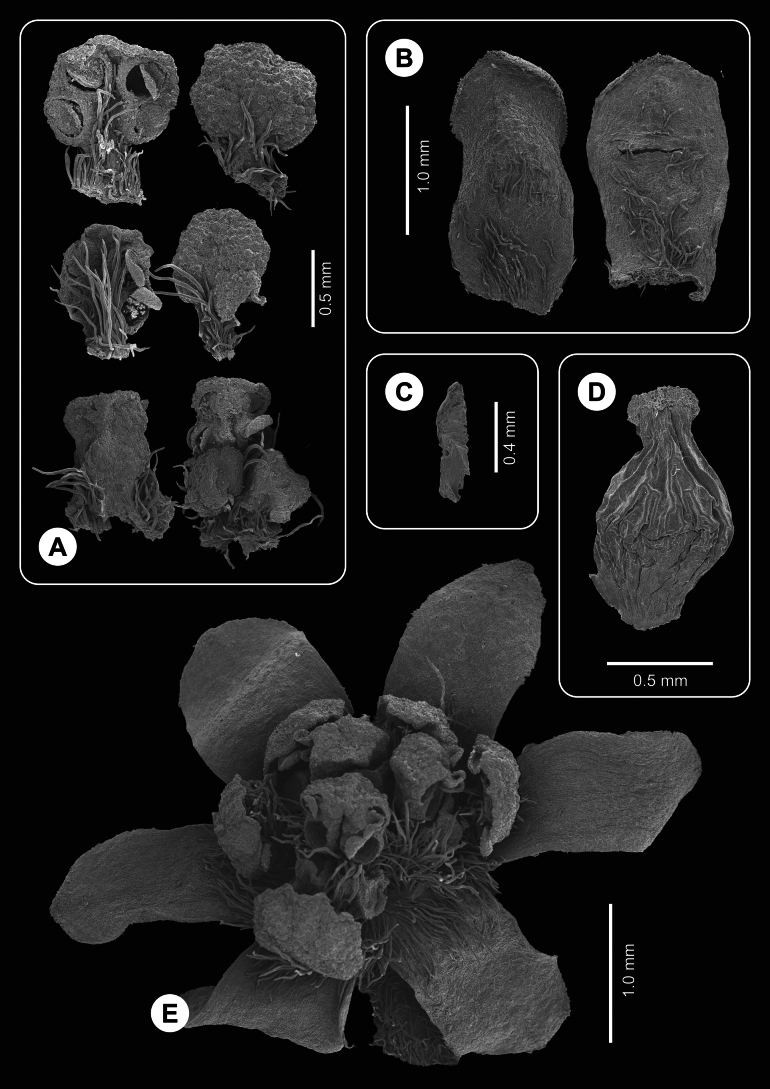
Flowers and floral parts of *Ocoteabella* (SEM photos) **A** stamens, adaxial and abaxial view, first to third whorl, in pairs from top to bottom **B** external view of tepals **C** staminode **D** pistil **E** general flower view (one whorl II stamen missing).

##### Distribution.

**Figure 4.**
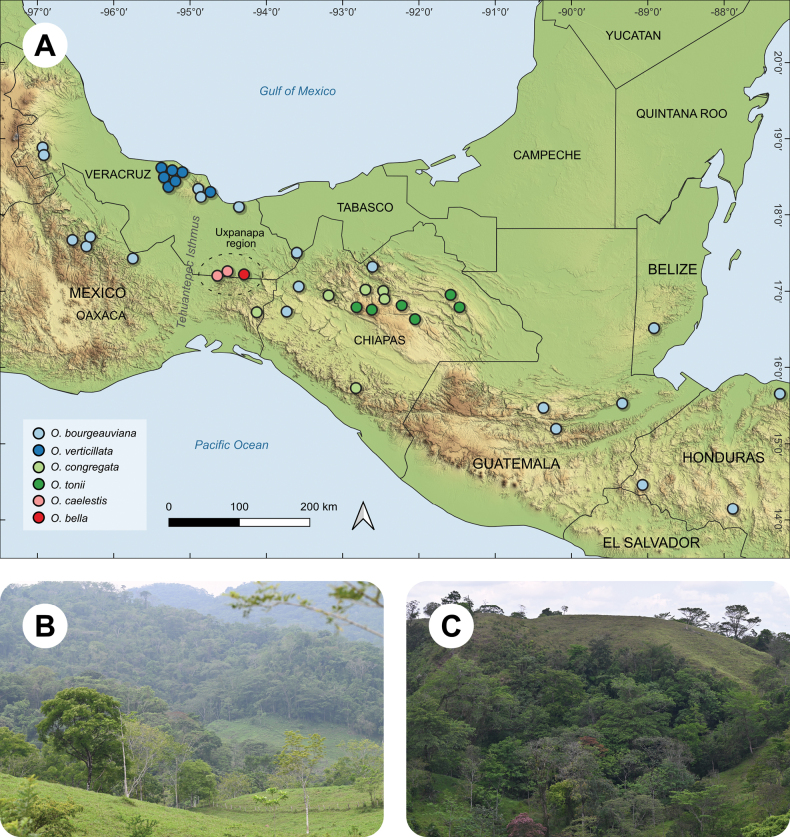
**A** distribution of the *Ocoteahelicterifolia* group species with clustered leaves **B** habitat of *Ocoteabella* at the type locality **C** habitat of *Ocoteacaelestis* at the type locality. Distribution localities are based on specimen records at the MEXU Herbarium.

*Ocoteabella* is known only from the Uxpanapa region in Veracruz, in Southern Mexico (Fig. 4).

##### Ecology and habitat.

*Ocoteabella* inhabits tropical rainforests on reddish clay-rich soils, with a mean annual temperature of 25 °C and a mean annual precipitation of 3450 mm. It has been collected at the base of north-facing hill slopes near a small stream.

##### Conservation status.

We currently lack the necessary information to assess definitively the conservation status of *Ocoteabella*. However, based on application of the criteria established by the IUCN (IUCN 2012, 2024), we conclude tentatively that the species is Critically Endangered [CR B2ab(ii,iii)]. Its area of occupancy (AOO) is 4.0 km, within the limits of Critically Endangered status under criterion B. The species is known from a single location (*sensu*IUCN 2024), also within the limit of the Critically Endangered status. In addition, the species appears to be rare. We found only one mature individual within an area of approximately 2 km^2^. Moreover, this species is not found within any protected natural areas, and it faces threats from deforestation and unsustainable practices such as logging, fires, and illegal settlements. Thus, we project that the ongoing loss of its habitat will induce a strong continuous decline of its AOO.

##### Notes.

Among the species of *Ocoteahelicterifolia* group, the reddish stamens of *Ocoteabella* are unique and distinguish it very well from any other species. It is an unusual character among neotropical Lauraceae. Additionally, the clustered leaves in this group seem restricted to northern Mesoamerica, ranging from Mexico to Honduras. Among the species with clustered leaves, the species most similar to *Ocoteabella* is *Ocoteacongregata* van der Werff, endemic to Chiapas, Mexico (Fig. 4). Vegetatively, *Ocoteacongregata* is distinguished from *Ocoteabella* by its smaller, broadly elliptical leaves, with rounded basis, obtuse to acute apex, and long petioles (Table 1). Additionally, *Ocoteacongregata* prefers montane forests (around 1500 m altitude) whilst *Ocoteabella* is restricted to lowland forests (200–300 m altitude).

#### ﻿ Ocotea caelestis

Taxon classificationPlantaeLauralesLauraceae

Ortiz-Rodr. & Reveles sp. nov.

582159CF-C138-555F-8638-77A0DB401961

urn:lsid:ipni.org:names:77356683-1

Figs 5 , 6

##### Type.

Mexico • Veracruz, Uxpanapa, 5 km south from Poblado 2 village, 17°11'28.4"N, 94°38'34.4"W, elev. 195 m, 1 May 2023, *Hurtado-Reveles L.* 1995 (holotype: MEXU; isotypes: NY, MO).

##### Diagnosis.

Similar to *Ocoteabourgeauviana* in having clustered leaves and a glabrous outer surface of the tepals, but differs from it in its smaller leaves and flowers, adaxial surface only pubescent at the base, tongue-shaped stamens with a sterile tip and with pubescent filaments (Table 1).

**Figure 5.**
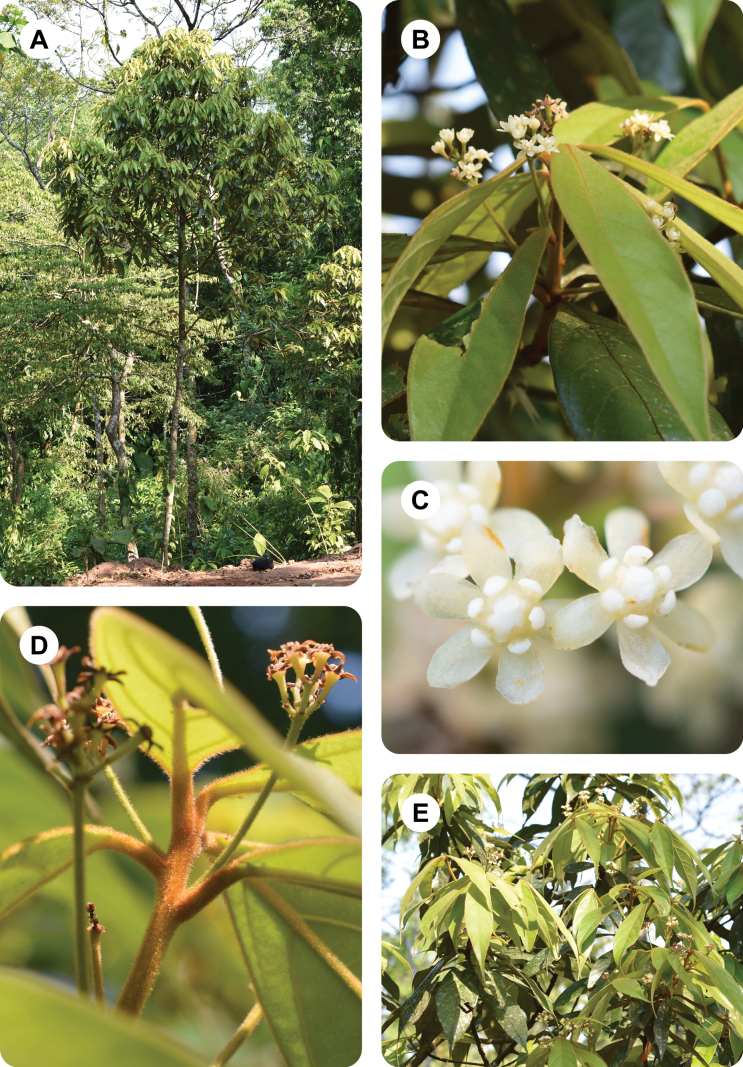
*Ocoteacaelestis* Ortiz-Rodr. & Reveles **A** general view of the tree **B** tip of flowering branch, showing leaf and inflorescence arrangement **C** flowers **D** apical part of recent growth showing the indument **E** branches with young and mature leaves.

##### Description.

***Trees***, evergreen, 4–8 m tall, 8–12 cm DBH; bark smooth to slightly striate; twigs terete, densely covered with erect and adpressed, golden trichomes, glabrous with age, terminal buds 5 × 5 mm, densely covered with erect and adpressed golden trichomes. ***Leaves*** aggregated to alternate-verticillate, clustered near the apex of branches, 2–5 × 7–18 cm, coriaceous, elliptic to lanceolate, base acute to attenuate, apex acute to caudate, upper surface smooth, glabrous, lower surface densely covered with erect golden trichomes, midrib slightly raised on the upper surface, prominently raised on the lower surface; secondary veins 5–8 on each side, slightly impressed above, prominently raised below; petiole swollen, 5–8 mm long, densely covered with erect and adpressed, golden trichomes. ***Inflorescences***, paniculate–cymose, axillary, 4–8 cm long, pedicels 1–3 mm long, the main axis, peduncles, pedicels and bracts densely covered with erect light–brown trichomes. ***Flowers*** bisexual, 4–5 mm in diam., tepals 6, subequal, ovate to elliptic, 2–3 × 1–2 mm, outer surface glabrous, inner surface with sparse, brown sericeous trichomes only near the base, stamens 9, in three whorls, white, ca. 1.5 mm long, filaments very short to anthers almost sessile, densely covered with small trichomes, anthers tongue-shaped, with a sterile tip which extends for ca. a third of the length of the total length of the anther, 4–locular, pollen sacs in two pairs above each other, inner stamens with two globose glands at the base, staminodes capitate, ca. 0.8 mm long, filament short and pubescent, pistil glabrous, ovary ovoid, 10 × 8 mm, style glabrous, ca. 0.5 mm long. ***Fruit*** unknown.

**Figure 6.**
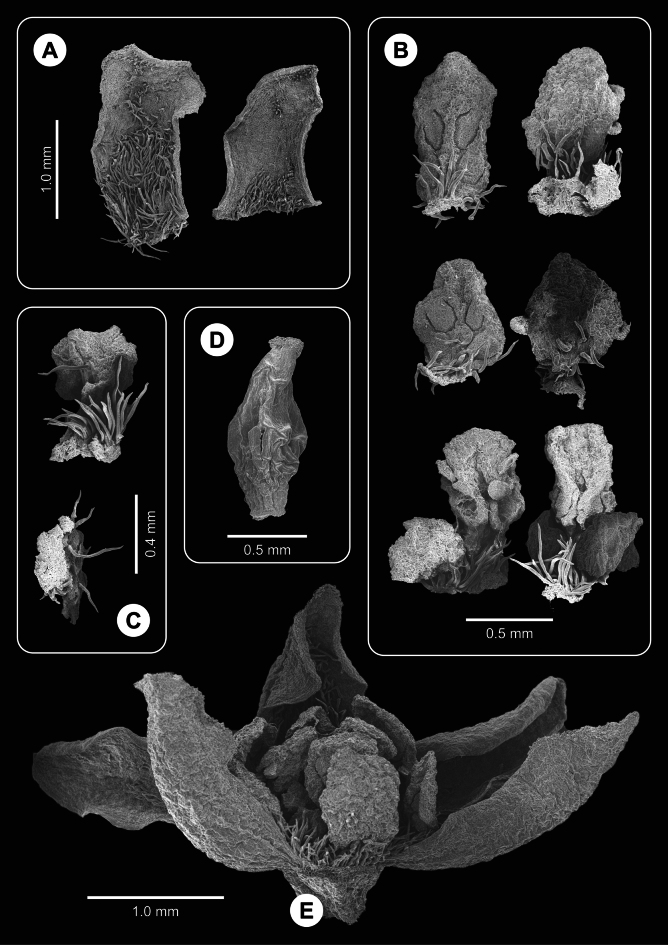
Flowers and floral parts of *Ocoteacaelestis* (SEM photos) **A** adaxial view of tepals **B** stamens, adaxial and abaxial view, first to third whorl, in pairs from top to bottom **C** staminode abaxial and side view **D** pistil **E** flower, lateral view, one tepal of first whorl (in front) removed.

##### Phenology.

This species has been collected with flowers from April to May.

##### Etymology.

The specific epithet refers to the ethereal appearance of this species (from the latin *caelestis*= heavenly appearance). The fine and dense golden indument in young leaves and twigs gives it a particular brightness during the first hours of the day. Moreover, inflorescences in living specimens notably rise above the flaccid leaves, which combined with their soft whitish color and almost glassy look gives them an ethereal appearance.

##### Distribution.

*Ocoteacaelestis* is known only from the Uxpanapa region in Veracruz, Southern Mexico (Fig. 4).

##### Ecology and habitat.

*Ocoteacaelestis* inhabits the tropical rainforests established on deep soils, with mean annual temperatures of 25 °C and mean annual precipitation of around 3250 mm. It occupies areas close to the hill ridges.

##### Conservation status.

Based on the criteria established by the IUCN (IUCN 2012, 2024), we conclude tentatively that the species is Critically Endangered [CR B2ab(ii,iii,v)]. Its area of occupancy (AOO) is 8.0 km^2^, within the limits of the Critically Endangered status under criterion B. The species is known from one location (*sensu*IUCN 2024), also within the limit of Critically Endangered status. Furthermore, only two adult individuals within an approximate area of 1 km^2^ have been found. The oldest collection near the type locality is more than 30 years old, suggesting that the species may persist in forest patches. Since the species is not found within any protected area, the increasing land use for livestock farming in the region is likely its main threat.

##### Additional specimens examined.

Mexico • Veracruz, Jesús Carranza, south from Poblado 2 village, 17°12'00"N, 94°38'30.0"W, elev. 200 m, 12 Apr 1987, Tom Wendt & H. Hernández G. 5622 (MEXU).

##### Notes.

Within the *Ocoteahelicterifolia* group, tongue-shaped stamens with a sterile tip have been reported only in *Ocoteabotrantha* Rohwer, *Ocoteasinuata* (Mez) Rohwer, *Ocoteatonii* (Lundell) van der Werff, *Ocoteaverticillata* Rohwer and now in *Ocoteacaelestis*. The clustered leaves relate *Ocoteacaelestis* to *Ocoteaverticillata*, from which it differs in its smaller leaves, with acute to cuneate basis, smaller flowers, outer surface of tepals glabrous, inner surface pubescent only at the base, and capitate staminodes shape (see Table 1).

Vegetatively, *Ocoteacaelestis* is very similar to *Ocoteabourgeauviana* (Mez) van der Werff and could hardly be separated in the absence of flowers. Flowering specimens of the two species can be easily distinguished. Flowers in *O.bourgeauviana* have trichomes distributed throughout the inner surface of the tepals (only at the base in *Ocoteacaelestis*), and its stamens are not tongue-shaped and do not have a sterile tip. Moreover, both species are allopatrically distributed (Fig. 4).

## ﻿Discussion

### ﻿Taxonomic novelties within a hyper-diverse region: Uxpanapa, Mexico

The isolated mountains of the Tehuantepec Isthmus in the Mexican states of Chiapas, Oaxaca, and Veracruz have long fascinated taxonomists due to their high level of endemism and species richness (Wendt 1987; Salazar 1999; Molina-Paniagua et al. 2023). In particular, the region of Uxpanapa, located between the states of Veracruz and Oaxaca, is considered a center of speciation and harbors many endemic species (Wendt 1987). Speciation processes in the Uxpanapa region may have been influenced by its role as a refuge for wet-tropical species during Pleistocene climatic fluctuations, as well as by its topographical complexity (Wendt 1987; Molina-Paniagua et al. 2023).

Only in the last two decades, several new plant species have been described from this region [e.g. Ceratozamia*dominguezii* Pérez-Farr. & Gut.-Ortega (Zamiaceae), *Desmopsisterriflora* G.E. Schatz, T. Wendt, Ortiz-Rodr. & Mart.-Vel. (Annonaceae), *Ixcheliauxpanapana* (T. Wendt) Wahlert & H.E. Ballard (Violaceae), *Lobelialithophila* Senterre & Cast.-Campos (Campanulaceae), *Magnoliauxpanapana* A. Vázquez, Padilla-Lepe & Gallardo-Yobal (Magnoliaceae), *Mortoniodendronuxpanapense* Dorr & T. Wendt (Malvaceae), *Peperomianopalana* G. Mathieu, *P.trichobracteata* G. Mathieu & T. Krömer & *P.xalana* G. Mathieu (Piperaceae), *Styraxuxpanapensis* P.W. Fritsch (Styraceae) (Dorr and Wendt 2004; Fritsch 2005; Senterre and Castillo-Campos 2008; Wahlert et al. 2015; Jimeno-Sevilla et al. 2018; Pérez-Farrera et al. 2021; Martínez-Velarde et al. 2023; Vázquez-García et al. 2024)]. An interesting pattern is the high level of divergence between species endemic to the Uxpanapa region and their closest relatives distributed outside this region (e.g. Senterre and Castillo-Campos 2008; Martínez-Velarde et al. 2023). Of course, the species described here fit this pattern well. *Ocoteabella* has flowers with red stamens and white petals, features absent in other species of the *Ocoteahelicterifolia* group. Also, only *Ocoteacaelestis* has the combination of clustered leaves with acute to cuneate basis and tongue-shaped stamens with a sterile tip.

Despite all of this, the Uxpanapa region is considered poorly surveyed, with intermittent exploration periods, old and unrepresentative collections and few specimens per species (Carvajal-Hernández 2018). Consequently, it is not surprising that new botanical explorations are leading to the discovery of new taxa. Considering the biological context, the Uxpanapa region’s future is alarmingly concerning. It is an area highly impacted by humans and vulnerable to climate change (Aguilar-López et al. 2016). Ongoing efforts to study and catalogue its biodiversity need to be strengthened, and effective conservation programs must be implemented (Carvajal-Hernández 2018).

## Supplementary Material

XML Treatment for Ocotea bella

XML Treatment for Ocotea caelestis

## ﻿Additional information

### Author ORCIDs

Leopoldo Hurtado-Reveles https://orcid.org/0000-0003-1660-2535

Andrés Ernesto Ortiz-Rodriguez https://orcid.org/0000-0003-4583-6701

### Data availability

All of the data that support the findings of this study are available in the main text.
